# Effects of polyphenols and lipids from *Pennisetum glaucum* grains on T-cell activation: modulation of Ca^2+^ and ERK1/ERK2 signaling

**DOI:** 10.1186/s12906-015-0946-3

**Published:** 2015-12-01

**Authors:** Abdelhafid Nani, Meriem Belarbi, Wided Ksouri-Megdiche, Souleymane Abdoul-Azize, Chahid Benammar, François Ghiringhelli, Aziz Hichami, Naim Akhtar Khan

**Affiliations:** University of Adrar, National Road n°06, Adrar, 01000 Algeria; Laboratory of Natural Products, Abou-bekr Belkaid University, Tlemcen, 13000 Algeria; INSERM U866, Université de Bourgogne, 21000 Dijon, France; Laboratoire des Plantes Extrêmophiles, Centre de Biotechnologie de Borj-Cédria, Hammam-lif, 2050 Tunisia

**Keywords:** Pearl millet, Polyphenols, Lipids, T-cells, Calcium

## Abstract

**Background:**

Pearl millet (PM), *i.e*., *Pennisetum glaucum*, is widely grown in Africa and known for its anti-oxidant and anti-hyperlipidemic properties.

**Methods:**

The* P. glaucum *grains were obtained from the region of Ouled Aïssa (South of Algeria).  We assessed the effects of phenolic compounds and lipids, extracted from seeds of *P. glaucum*, on rat lymphocyte proliferation, activated by phorbol 12-myristate 13-acetate and ionomycin. In order to explore signaling pathway, triggered by these compounds, we assessed interleukin-2 (IL-2) mRNA expression and extracellular signal-regulated kinase-1/2 (ERK1/ERK2) phosphorylation. Finally, we determined increases in free intracellular Ca^2+^ concentrations, [Ca^2+^]i, by employing Fura-2/AM in rat lymphocytes.

**Results:**

The composition of *P. glaucum* grains in polyphenols was estimated to be 1660 µg gallic acid equivalents (GAE)/g. Lipids represented 4.5 %, and more than 72% of the fatty acids belonged to unsaturated family. Our investigation showed that both lipid and phenolic compounds inhibited mitogen-induced T-cell proliferation. Compared with phenolic compounds, lipids exerted weaker effects on ERK-1/ERK2 phosphorylation and Ca^2+^ signaling in mitogen-activated T-cells.

**Conclusion:**

We conclude that the immunomodulatory effects of *P. glaucum * could be contributed by its phenolic and lipid contents.

## Background

Current recommendations from international health and nutritional organizations, like Food and Drug Administration (FDA), include an increase in the consumption of high-bran cereals because of their potential benefits on human health [1]. Millets are cereals that have been cultivated for more than 3500 years in all Sahelian Africa and tropical countries of Western Africa. Indeed, millets are extremely resistant to dryness and well adapted to manure-poor soil [2]. Millet refers to a number of different species, and all of them are small-grained and annual cereal grasses [3].

Millet grains have been shown to exert beneficial effects in health and disease [4–6]. Lee et al. [4] reported that foxtail and proso millet decreased plasma triglycerides in hyperlipidemic rats. Shobana et al. [5] reported that feeding a diet containing 20 % finger millet decreased hyperglycemia and its associated complications in streptozotocin-induced diabetic rats. In clinical studies, finger millet exerted anti-hyperglycemic effects in diabetic patients [6]. The beneficial effects of pearl millet (PM) have not been well studied except a few available reports that have shown anti-oxidant activity because of its high contents in polyphenols [7]. Luteolin, a flavone present in millets, has been reported to exert antioxidant, anti-inflammatory and cancer-preventive properties [8, 9]. Van Rensburg [10] and Chen et al. [11] reported that populations consuming cereals including millet had lower incidences of esophageal cancer compared to those consuming wheat or maize. Nani et al. [12] have shown that streptozotocine (STZ)-induced diabetic Wistar rats fed with pearl millet-enriched diet underwent a significant curtailment in glycaemia and an improvement of body weight. Regarding its chemical composition, pearl millet has been attributed to having several health promoting abilities: anemia, constipation, cancer, and diabetes [13].

As far as immune system is concerned, cereal consumption has been reported to exert immune-modulating activities. Recent studies have shown that cereals contain a wide range of phenolic compounds [14–17]. The immunomodulatory effects of polyphenols have drawn considerable attention in recent years [18–20]. González et al. [21] have reported that flavonoids and related polyphenolic compounds possess anti-inflammatory activity. Furthermore, the mechanisms of action of other polyphenols, e.g., resveratrol, curcumin, genistein and epigallocatechin, in the modulation of immune system and the secretion of pro-inflammatory mediators have been reviewed [22]. In addition to dietary polyphenols, a great attention has been paid to dietary lipids which are able to modulate inflammatory status, depending on their fatty acid content and composition [23–25]. *In vitro* and *in vivo* studies have shown that fatty acids modulate a number of lymphocyte functions [25], including proliferation [26], cytokine release [27], and mitogenic signaling [28].

Pearl millet (PM), i.e., *Pennisetum glaucum*, is the most widely grown species in Africa [29], and it constitutes the daily basic food for 50 million inhabitants of the Sahel [2]. PM contains several compounds including lipids and polyphenols that could modulate immune system. To our knowledge, no study concerning the effects of PM polyphenolic or lipidic fractions on the modulation of immune system is available. We, therefore, investigated the effect of phenolic compounds and lipids, extracted from PM, on T-cell activation. Since an increase in free intracellular Ca^2+^ concentration, [Ca^2+^]_i_ and mitogen-activated protein kinase (MAPK) phosphorylation, are the part of early events of T-cell activation, it was thought worthwhile to elucidate the effect of PM lipids and phenolic extracts on Ca^2+^ and MAPK signaling in T-cells.

## Methods

### Materials

Grains of pearl millet, *Pennisetum glaucum*, obtained from the region of Ouled Aïssa (174 km in the North of Adrar city and 70 km to the North-West of Timimoun, Algeria), were used in this study. Wistar rats were obtained from Janvier Elevage (Le Genest-st-isle, France). RPMI 1640 medium and L-glutamine were purchased from Lonza Verviers SPRL (Verviers, Belgium). Fura-2 AM was procured from Life Technologies (France). Anti-Phospho-p44/42 mitogen-activated protein kinase (MAPK, Erk1/2) and anti-p44/42 MAPK (Erk1/2) were obtained from Cell Signaling (France). All other chemicals were purchased from Sigma (USA).

The general guidelines for the care and use of laboratory animals, recommended by the council of European Economic Communities, were followed. The experimental protocol was approved by the Regional Ethical Committee (Dijon).

### Extraction and determination of phenolic compounds

The plant was recognized by a botanist (Pr Benabadji Nouri, Université Aboubekr Belkaïd, Tlemcen) of the Herbarium Center of the Faculty of Pharmacy (Tlemcen) which contained the voucher specimen (PM 1681). PM phenolic extracts were obtained according to the method of Liyana-Pathirana and Shahidi [30] with slight modifications. Briefly, 2 g of PM grain powder were extracted two times (2 h for each extraction) with 40 ml of methanol–acetone–water (7:7:6, v/v/v) at room temperature (25 ± 2 °C) with constant stirring. The mixtures were centrifuged (20 min at 4000 g) and supernatants were collected and subjected to extraction with an equal volume of hexane to eliminate lipids.

Total phenolic contents in plant extracts were determined by Folin-Ciocalteu method [31] as described by Miliauskas et al. [32]. Briefly, 0.5 ml polyphenol extract was reacted with 2.5 ml of Folin-Ciocalteu reagent (0.2 mol/l) for 4 min, then 2 ml saturated sodium carbonate solution (75 g/l) was added into the reaction mixture. After 2 h incubation at room temperature, the absorbance at 760 nm was determined. The content of phenolic compounds was determined with reference to standard curve determined with gallic acid. The content of phenolic compounds was expressed as μg gallic acid equivalents (GAE)/g dry matter (μg GAE/g).

Phenolic extract (20 μl) was analyzed by HPLC (Model Agilent Technologies 1260, Germany) with reverse phase Zorbax Eclipse XDB-C18 column (4.6 × 100 mm) and a diode array UV-detector (operating at 280 nm). The gradient mobile phase was composed of two solvents: A and B. Solvent A was methanol and solvent B was 0.1 % formic acid (v/v). Phenolic acid separation was achieved using a 35 min linear solvent gradient at a flow rate of 0.4 ml/min, as follows: 0 min 90 % B, 5 min 80 % B, 10 min 70 % B, 15 min 50 % B, 20 min 30 % B, 25 min 10 % B, 30 min 50 % B, 35 min 90 % B. PM phenolic compounds were identified with reference to retention time of authentic standards and quantified on the basis of their peak area. Standard phenolic compounds used were: p-coumaric, chlorogenic, ferulic, gallic, trans-hydroxycinnamic, syringic acid, ellagic acids, quercetin and apigenin.

### Lipid extraction

Lipids were extracted according to the method of Hara and Radin [33]. In order to avoid oxidation, all solvents contained 0.01 % butylated hydroxytoluene (BHT). Briefly, 1 g of millet grain powder plus 20 μg of internal standard (C19:0) were blended with 8 ml isopropanol and heated at 80 °C for 5 min. After cooling to room temperature, the blended grain powder was crushed in 12 ml hexane. The mixture was briefly centrifuged, and then the upper phase was collected. For complete recovery, the pellet was re-extracted with 9 ml hexane and 2 ml isopropanol, and then the extract was combined with the upper phase of the previous step. To remove non-lipid fraction, the extract was partitioned into an upper hexane phase by the addition of aqueous sodium sulphate 6.5 % (0.5/1 : v/v). The upper phase, lipid extract, was transferred into a new tube, dried under a stream of nitrogen and stored at −20 °C until fatty acid analysis by gas–liquid chromatography (GLC).

### Preparation of fatty acid methyl esters (FAMEs)

In brief, 0.1 ml of hexane, containing lipid extract, was transferred into screw cap tubes and dried under nitrogen, then 1 ml of methanolic NaOH (0.5 N) was added into tubes, and heated at 80 °C during 20 min. After cooling at 4 °C, 2 ml of boron trifluoride-methanol solution (BF3) were added, and the methylation was performed at 80 °C for 20 min. After cooling-down in ice, 2 ml of NaCl (35 %) plus 2 ml of hexane were added into the tubes. After vigorous agitation and centrifugation (1200 g/5 min), the upper phase containing fatty acid methyl esters was transferred into new tubes, and analyzed by gas − liquid chromatography (GLC).

### Gas liquid chromatography (GLC)

GLC was performed in a Packard Model 417 gas–liquid chromatograph, equipped with a flame ionization detector and a 30-m capillary gas column coated with carbowax 20 M. The analysis conditions were as follows: oven temperature was 85 °C/1 min, increased to 150 °C at 30 °C/min, then increased at 4 °C/min to 210 °C. Helium was used as carrier gas, with a flow rate of 0.4 ml/min. Analysis of fatty acid peaks was achieved with reference to the internal standards (Nu-Chek-Prep, Elysian, MN) by using DELSI ENICA 31 (Delsi Nermag, Rungis, France). The fatty acid levels were expressed as g/100 g of total fatty acids.

### Isolation and preparation of splenic T-cells

Fresh splenocytes were harvested from Wistar rat spleens under aseptic conditions. The removed spleens were immediately transferred to the petri dishes, containing RPMI-1640 complete medium (RPMI 1640 medium supplemented with 10 % foetal calf serum, 2 mM L-glutamine, 100 U/ml of penicillin, and 100 μg/ml of streptomycin and 25 mM HEPES) . Spleens were teased apart using a wire gauge. After lysis of red blood cells, with Red Cell Lysing Buffer (Sigma, USA) and centrifugation (200 × g, 5 min), the pellet was resuspended in RPMI-1640 complete medium, and placed into a sterile petri dish for 1 h at 37 °C, in a humidified chamber containing 95 % air and 5 % CO_2_, to remove the macrophages by adherence. T lymphocytes were isolated by panning [34]. In brief, the unadhered cells were decanted, centrifuged (200 × g, 5 min) and transferred to the petri dishes that were previously coated with anti-rat IgG (5 μg/ml) overnight at 4 °C. Hence, selective depletion of B lymphocytes was accomplished because they adhered to the substratum of the petri dishes. After an incubation of 1 h at 4 °C, the T-lymphocyte–rich supernatant was decanted and centrifuged (200 × *g*, 5 min) twice with RPMI-1640 complete medium. This technique provided us with an enriched (99 %) T-cell population as verified by cyotofluorimetry (not shown). Cell numbers were determined by hemocytometer.

### T-cell proliferation assay

The proliferation of splenic T-cells in response to PM lipids and polyphenols was assessed according to the method of Bonin and Khan [35] with slight modification. Splenic T-cells were resuspended in RPMI complete medium and plated into 96-well microplates at the concentration of 5 × 10^5^ cells/well. Lipid and polyphenol extracts obtained from pearl millet, were solubilized in ethanol, and added to cells, final ethanol volume was below 0.1 % (v/v), 1 h before their activation with PMA (50 nM) and ionomycin (500 nM). After 48 h of treatment, T-cell proliferation was measured by Cayman’s WST-8 cell proliferation assay kit (Cayman Chemical, USA). The stimulation index (SI) was calculated as follows: SI = optical density (450 nm) of stimulated cells/optical density (450 nm) of unstimulated cells × 100.

### Cell preparation for western blot analysis

Splenic T-cells were serum starved for 6 h in RPMI 1640 medium without serum. Then, splenic T- cells (5 × 10^6^/ml) were pre-incubated for 5 min with either PGL or PGPC or vehicle before stimulation with PMA (200 nM) for an additional 30 min, according to Nel et al. [36]. Incubation was stopped by centrifugation, and cell pellets were washed twice with PBS and resuspended in 50 μl of lysis buffer (HEPES, 20 mM, pH 7.3; EDTA, 1 mM; EGTA, 1 mM; NaCl, 0.15 mM; Triton X-100, 1 %; glycerol, 10 %; phenylmethylsulfonyl fluoride, 1 mM; sodium orthovanadate, 2 mM; antiprotease cocktail, 2 μl in 1 ml of buffer). After centrifugation (2500 g for 1 min), the protein in the supernatant was quantified with the bicinchoninic acid (BCA) assay (Thermo Fisher Scientific, France) and either used immediately for Western blot detection or stored at −80 °C.

### Western blot detection of phosphorylated MAP kinases

Denatured proteins (60 μg) were separated by SDS-PAGE (10 %) and transferred onto polyvinylidine difluoride membranes, and immunodetection was performed by using rabbit antibodies raised against phosphorylated or non-phosphorylated P44/P42 MAPK. Primary antibodies were detected with a horse radish peroxidase conjugated mouse anti-rabbit antibody and visualised using an ECL Kit (Merck Millipore) on Bio-Rad ChemiDoc XRS^+^ system. Densitometric analysis was performed on Bio-Rad Image Lab Software (version 4.1).

### RNA isolation and real time quantitative PCR

Cells were cultured as described above in the presence of either PGL or PGPC extracts and stimulated with anti-CD3 antibodies for 2 h [37, 38]. Total RNA was extracted using TRIzol Reagent and underwent DNase treatment using the RNase-free DNase Set (Life Technologies). 500 ng of total RNA was reverse transcribed with Super script II H-reverse transcriptase (Life Technologies) using oligo (dT) according to the manufacturer’s instructions. Real time PCR was carried out on the iCycler iQ real time detection system and amplification was undertaken by using SYBR® Green PCR Master Mix (Life Technologies) as described elsewhere [39]. Oligonucleotide primers were as follow: beta-actin forward: 5′-ATGATA TCGCCGCGCTCGTCGTC-3′, beta-actin reverse 5′-AGGTCCCGGCCAGCCAGGTCCAG-3′; IL-2 forward 5′ CACTAATTCTTGCACTTGTCAC-3′, IL-2 reverse 5′- CTTCTTGGGCATGTAAAACT-3′. Relative quantification of IL 2 mRNA was determined by ΔΔCt method as follows: ΔCt = Ct of IL-2 - Ct of beta actin. ΔΔCt = ΔCt of treated cells - ΔCt control cells. Relative quantity (RQ) was calculated as follows: RQ = (1 + E)^-(ΔΔCt)^ .

### Measurement of free intracellular Ca^2+^concentrations; [Ca^2+^]_i_

Splenic T-cells (2 × 10^6^ cells/ml) were washed with phosphate-buffered saline, pH 7.4, and then incubated with Fura-2/AM (1 μM) for 60 min at 37 °C in loading buffer containing: 110 mM, NaCl; 5.5 mM, KCl; 25 mM, NaHCO_3_; 0.8 mM, MgCl_2_; 0.4 mM, KH_2_PO_4_; 0.33 mM, Na_2_HPO_4_; 20 mM, HEPES; 1.2 mM, CaCl_2_, and the pH was adjusted to 7.4. After loading, the cells were washed three times (720 g × 10 min) and remained suspended in the identical medium. The fluorescence intensities were measured in the ratio mode in the PTI spectrofluorometer at 340 and 380 nm (excitation filters) and 510 nm (emission filters). The cells were continuously stirred throughout the experiment. The intracellular concentrations of free Ca^2+^, [Ca^2+^]i, were calculated by using the following equation: [Ca^2+^]i = Kd × (R-Rmin)/(Fmax-F)(Sf2/Sb2). A value of 224 nM for Kd was added into the calculations. R_max_ value was obtained by the addition of ionomycin (5 μM) and R_min_ value was obtained by the addition of MnCl_2_ (2 mM), Triton X-100 (0.1 %) and EGTA (24 mM).

For experiments conducted in the absence of external calcium (0 % Ca^2+^), CaCl_2_ was replaced by 1 mM EGTA in the buffer [40]. All test molecules were added in small volumes with no interruption in recordings.

### Statistical analyses

Results are shown as mean ± SD (standard error deviation) for a given number of experiments (n). Data were analysed by using Statistica (4.1 version, Statsoft, Paris, France). The significance of differences between mean values was determined by one-way ANOVA, followed by Fisher’s least-significant-difference (LSD) test. Differences with *p* < 0.05 were considered to be significant.

## Results

### Phenolic acid and lipid composition of PM grains

The total content of phenolic compounds in PM grains was estimated to be 1660 μg GAE/g. Table 1 shows that *p*-coumaric acid represents 81 %, and ferulic acid represents 12 % of the total phenolic compounds.

**Table 1:** Phenolic acid composition of PM grains

Phenolic compounds	μg/g sample (dry weight)
Gallic acid	15.351
Chlorogenic acid	16.074
Syringic acid	7.380
*p*-Coumaric acid	1350.884
Ferulic acid	199.562
Hydroxycinnamic acid	41.330
Ellagic acid	14.4364
Quercetin	5.904
Apigenin	9.078

Total lipids were estimated to be 4.5 % (Table 2). Alpha-linoleic acid (18:2n-6) was the most abundant fatty acid (44.95 %), and oleic acid (18:1 n-9) was the second most abundant fatty acid (24.88 %). The proportion of unsaturated fatty acids was estimated to be 72 % (Table 2).

**Table 2:** Fatty acid composition (% of total FA) of *Pennisetum glaucum*

Fatty acids		Content (%) in total oil
Palmitic	16:0	20.13 ± 0.16
Palmitoleic	16:1	0.52 ± 0.04
Stearic	18:0	5.11 ± 0.19
Oleic	18:1	24.88 ± 0.19
Linoleic	18:2n-6	44.95 ± 0.32
Linolenic	18:3n-3	3.03 ± 0.03
Arachidic	20:0	0.90 ± 0.02
Gonodoic	20:1	0.25 ± 0.01
Behenic	22:0	0.23 ± 0.01

### PGL and PGPC decrease T-cell proliferation

**Fig. 1:**
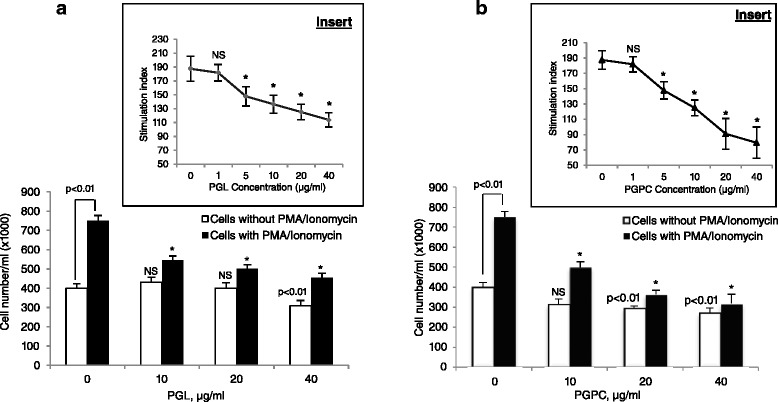
Effect of PGL and PGPC on T cell proliferation. The cells (5 × 10^5^ cells/ml) were stimulated with different concentrations of the extracts as described in Methods. Cell number were determined with a hemocytometer. Inserts show the stimulation index (SI) of T-cell proliferation in response to PGL (left panel) and PGPC (right panel). Data represent means ± SD (*n* = 6). *p* < 0.01 as compared to cells without PGL or PGPC, *represents *p* < 0.001 as compared to PMA + Iono-stimulated T-cells. NS = insignificant differences. *p* values were obtained by one-way ANOVA, followed by Fisher’s LSD test

Figure 1a shows the effects of increasing concentration of PGL on T-splenocyte proliferation either in the presence or absence of mitogens (PMA + Iono). We observed that PGL until 20 μg/ml concentration exerted no significant effect on basal splenic T-cells proliferation. However, PGPC at 20 μg/ml decreased basal T-cells proliferation. We also observed that both PGL and PGPC curtailed, in a dose-dependent manner, T-cell proliferation induced by PMA + Iono. PGPC exerted more inhibitory effect on T-cell proliferation than PGL (Fig. 1b).

### PGL and PGPC diminish PMA-induced ERK1/ERK2 activation

**Fig. 2:**
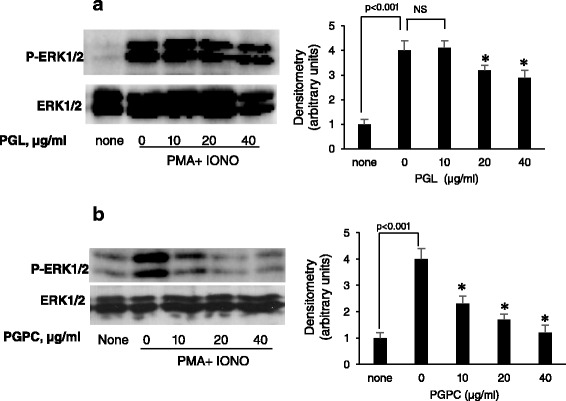
Effects of PGL and PGPC on PMA + Iono-stimulated ERK1/ERK2 phosphorylation in splenic T-cells. Data were quantified by densitometry and expressed as phosphorylated /non phosphorylated ERK1/ERK2 ratio. Splenic T-cells (5 × 10^6^ cells/ml), before determination of MAP kinase phosphorylation, were incubated for 6 h in RPMI 1640 medium without serum, and treated with increasing concentrations (0 to 40 μg/ml) of PGL in (**a**) and PGPC in (**b**). After 5 min of incubation, cells were stimulated with PMA (50 nM) and Iono (500 nM) for another 30 min at 37 °C. Cells were lysed and phosphorylated MAP kinases were detected performed as described in Materials and Methods. Results are expressed as arbitrary units in bar graphs. *Represents *P* < 0.001 as compared to PMA + Iono-stimulated T-cells in the absence of PGL (**a**) and PGPC (**b**). NS = insignificant differences. *p* values were obtained by one-way ANOVA, followed by Fisher’s LSD test

Figure 2 shows that both PGL and PGPC dose dependently diminished PMA-induced ERK1/2 phosphorylation in splenic T-cells. PGPC completely blocked MAP kinase phosphorylation even at a low concentration (10 μg/ml) (Fig. 2b), whereas the effects of PGL was weaker than that of PGPC. (Fig. 2a).

### PGL and PGPC decrease IL-2 mRNA expression

**Fig. 3:**
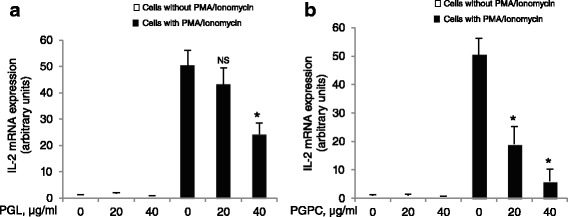
PGL and PGPC modulate IL-2 mRNA expression. Splenic T-cells (5 × 10^5^ cells/ml) were incubated with increasing concentrations (0 to 40 μg/ml) of PGL (**a**) and PGPC (**b**), and stimulated with anti-CD3 antibodies for 2 h. Each value represents the mean of three determinations. * Represents *P* < 0.001 as compared to PMA + Iono-stimulated T-cells. NS = insignificant differences. The *p* values were obtained by one-way ANOVA, followed by Fisher’s LSD test

Our results show that PGL and PGPC exerted no effect on basal expression of IL-2 mRNA in splenic T-cells*.* However, both PGL and PGPC extracts diminished, in a dose-dependent manner, PMA + Iono-induced IL-2 mRNA expression (Fig. 3). As observed for T-cell proliferation and ERK1/2 phosphorylation, PGPC exerted more important inhibitory effect than PGL on IL-2 mRNA expression.

### PGL and PGPC induce increases in [Ca ^2+^]i in splenic T-cells

**Fig. 4:**
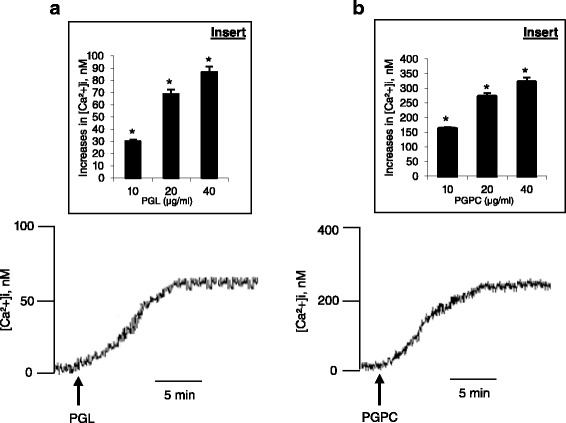
Ca^2+^ signaling modulation by PGL and PGPC in splenic T-cells. Cells (2 × 10^6^/ml) were loaded with the fluorescent probe, Fura-2/AM, as described in Methods. The arrow head indicates the time when PGL or PGPC were added into the cuvette without interruptions in recordings. Figures show the single traces of observations which were reproduced independently (*n* = 6). Inserts show the increase in [Ca^2+^]i evoked by the increasing concentrations (10 to 40 μg/ml) of PGL (**a**) and PGPC (**b**). *Represents *P* < 0.001 as compared to control (untreated cells). NS = insignificant differences. The *p* values were obtained by one-way ANOVA, followed by Fisher’s LSD test

**Fig. 5:**
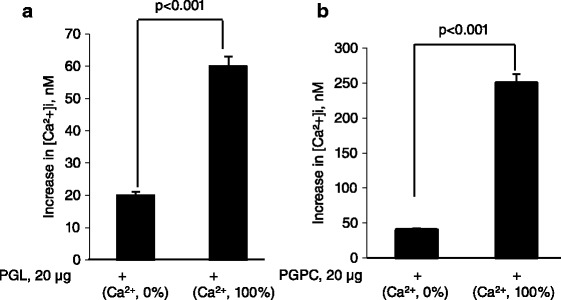
Ca^2+^ signaling modulation by PGL (**a**) and PGPC (**b**) in splenic T-cells in 0 % Ca^2+^-buffer and 100 % Ca^2+^-buffer. PGL and PGPC-evoked increases in [Ca^2+^]i are curtailed in 0 % Ca^2+^-buffer in T-cells. Cells (2 × 10^6^/ml) were loaded with the fluorescent probe, Fura-2/AM, as described in Methods. *Represents *P* < 0.001 as compared to [Ca^2+^]i increases in 0 % Ca^2+^-buffer. The *p* values were obtained by one-way ANOVA, followed by Fisher’s LSD test

Figure 4 shows that both PGL and PGPC evoked a dose-dependent increase in [Ca^2+^]i in splenic T-cells; however, the increase in [Ca^2+^]i triggered by PGPC was significantly higher than that triggered by PGL (Fig. 4). In order to assess the origin of Ca^2+^ mobilized by lipids and phenolic compounds, we conducted experiments in the absence (0 % Ca^2+^) and presence (100 % Ca^2+^) of Ca^2+^ in the extracellular medium. Figure 5 shows that the PGPC and PGL induced a weak and discrete increase in [Ca^2+^]i in 0 % Ca^2+^ medium as compared to that induced in 100 % Ca^2+^ medium.

**Fig. 6:**
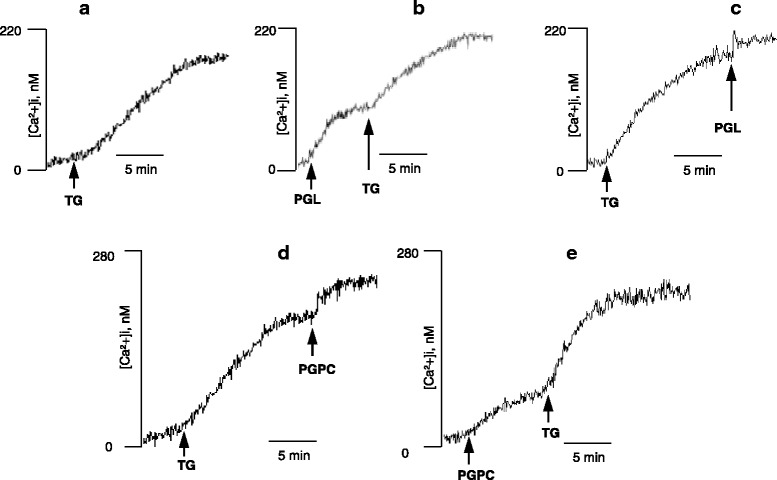
Effects of thapsigargin (TG) on PGL and PGPC-induced rise in [Ca^2+^]i in lymphocytes that were tretaed as follows : TG (**a**), PGL followed by TG (**b**), TG followed by PGL (**c**), TG followed by PGPC (**d**, and PGPC followed by TG (**e**). Cells (2 × 10^6^/ml) were loaded with the fluorescent probe, Fura-2/AM, as described in Methods. The arrow head indicates the time when 20 μg/ml of PGL, PGPC, or TG (5 μM) were added into the cuvette. The figure shows the single traces of observations reproduced independently (*n* = 6)

As the polyphenolic and lipidic extracts were able to induce increases in [Ca^2+^]i even in the absence of external calcium, it was thought worthwhile to examine the nature of intracellular stores involved in this rise in [Ca^2+^]i. We used thapsigargin, known to induce increase in [Ca^2+^]i by inhibiting the endoplasmic reticulum Ca^2+^-ATPase [41, 42]. Figure 6 illustrates that thapsigargin alone triggered a calcium peak, and addition of PGL or PGPC after thapsigargin or vice versa evoked additive effects on the increases in [Ca^2+^]i in these cells.

## Discussion

Millet grains, widely consumed in many areas of Asia, Africa and Latin America, have been shown to exert several beneficial effects in health and disease [5]. Among millets species, pearl millet (PM) has been the least studied. In this study, we examined the effects of polyphenols and lipids, extracted from PM, on T-cell proliferation. We investigated the involvement of calcium and MAP kinase signaling in this process.

Most of the millet species contain phenolic compounds, which are detected in the pericarp, testa, aleurone layer, and endosperm [43]. In our study, the total polyphenol content of the PM extract was estimated to be 1660 μg GAE/g, in agreement with other studies in which the values ranged from 1387 to 2580 μg GAE/g [44, 45]. Similarly to the finding of Shahidi and Chandrasekara [46], we observed that PM grains contained principally *p*-coumaric acid and ferulic acid. In addition, apigenin, a flavonoid, was detected in PM grains [46].

It is well established that, except finger millet, millet species have higher lipid contents, ranging from 3.5 % to 5.2 %, than other cereals [46]. In our investigation, total lipids were estimated to be 4.5 % in PM grains. Indeed, Ragaee et al. [44] found that PM had the highest content of lipids (4.2 %) compared to wheat flours and other cereal whole grains. The high content of lipids in PM grains might be due to the presence of embryo in which lipids are concentrated. Daniel et al. [47] had reported that PM oil yielded three fatty acids as major components. Hence, alpha-linoleic acid amounted to be 45.6 % followed by oleic acid (28.5 %) and palmitic acid (20.6 %), whereas linolenic and stearic acids were the minor fatty acids. In our samples, we obtained a high amount of both linolenic and stearic acid (3.03 % and 5.11 %, respectively).

T-lymphocytes represent a fundamental component of the adaptive immune response. The lymphocyte transformation assay is an important tool to measure, *in vitro,* mitogen-induced lymphocyte proliferation [48, 49]. Following T-cell receptor (TCR) engagement, one of the early events in T-cell activation is the phosphorylation of tyrosine kinases and the generation of inositol 1,4,5-triphosphate (IP3), leading to the release and influx of Ca^2+^, and the rise in cytoplasmic Ca^2+^ concentration [50]. The rise in [Ca^2+^]i activates via calcineurin induces IL-2 gene expression [51]. To our knowledge, the present report is the first study assessing the immunomodulatory effects of PM polyphenols and lipids. PM extracts were rich in apigenin, *p*-coumaric acid and other phenolic acids. Apigenin has been shown to inhibit T-cell proliferation [52], without exerting any toxic effect [53]. Interestingly, *p*-coumaric acid has been reported to exert anti-cancer [54], anti-mutagenic [55], and anti-inflammatory activities [56]. PGPC strongly inhibited T-cell proliferation and IL-2 mRNA expression. Other investigators have also reported that plant polyphenols inhibited proliferation and IL-2 production in human lymphocytes [39, 57, 58]. Gao et al. [59] reported that resveratrol, a stilbene, inhibited the proliferation and IL-2 and interferon (IFN)-γ production by splenic lymphocytes. Kaempferol, a flavonoid, was able to reduce IFN-γ and IL-2 production by murine T-cells [60]. Curcumin, that gives rise mainly to ferulic acid and vanillin, also inhibited IL-2-induced T-proliferation of splenic cells [61].

PGL also, to a lesser extent than PGPC, inhibited T-cell proliferation. In PGL, n-3:n-6 ratio was estimated to be 1:14 which is very close to the recommended ratio (1:10), reported by Ma et al. [62]. The inhibitory effect of lipid extract of PM may be attributed to linoleic acid, an n-6 fatty acid. Linoleic acid (18: 2n-6) which represents 44.95 % of total fatty acids in PGL, could be involved in T-cell immunosuppression. Indeed, Liu et al. [63] had reported that linoleic acid inhibited IL-2 mRNA expression and, consequently, lymphocyte proliferation. Another study has shown that linoleic acid was a potent inducer of cell death in human peripheral blood lymphocytes. The mechanism of action of linoleic acid on cell apoptosis involved alterations in mitochondrial transmembrane potential and ROS production [64]. Similarly, linolenic acid (18: 2n-3) could be involved in T-cell immunosuppression. Indeed, Denys et al. [65] have shown that n-3 fatty acids inhibited mitogen-induced nuclear translocation of NF-κB and IL-2 mRNA expression in Jurkat T-cells. The inhibitory effect of PGPC and PGL on T-cell proliferation could be mediated by their capacity to reduce IL-2 mRNA expression. In fact, the transition of T-cells via S phase of cell cycle is associated to the expression of IL-2 mRNA. The newly synthesized IL-2 acts in an autocrine manner in order to assure the T-cell cycle progression. Furthermore, inhibition of IL-2 production is associated with cell cycle arrest [66].

Mitogen-activated protein (MAP) kinases including the extracellular signal-regulated kinase-1/2 (ERK1/ERK2) have been shown to play a critical role in the events leading to increased IL-2 production in mammalian cells [36, 67]. Both PGL and PGPC dose-dependently diminished PMA-induced ERK1/ERK2 phosphorylation in splenic T-cells; however, the inhibitory effect of PGPC was more pronounced. Our results agree with the observations of Neuhaus et al. [68] who had demonstrated that the phosphorylation of ERK1/ERK2 was inhibited by epigallocatechin-3 gallate (EGCG). Similarly, the treatment of ECV304 cells with ferulic acid, a major phenolic acid in pearl millet, inhibited both cell proliferation and ERK1/ERK2 phosphorylation [69]. Besides, the PM lipid contents might be responsible for the inhibition of MAPK phosphorylation as reported previously [65].

We examined the actions of PGL and PGPC on the increases in [Ca^2+^]i in T-cells. In the presence of 100 % Ca^2+^, PGPC and PGL induced high increases in [Ca^2+^]i, suggesting that Ca^2+^ influx plays a major role in the increase in [Ca^2+^]i evoked by theses extracts. The kinetic study of PGL and PGPC-induced Ca^2+^ mobilization showed that these compounds produced a sustained increase in [Ca^2+^]i. We also noted that PGPC induced stronger and more important increases in Ca^2+^ (around 6-fold), compared to PGL. These sustained [Ca^2+^]i increases are correlated with the immunosuppressive effects of PM extracts, as reported for the prickly pear phenolic compounds [40].

To ascertain the nature of the intracellular Ca^2+^ pool mobilized by lipids and phenolic compounds, thapsigargin, an inhibitor of Ca^2+^-ATPase of endoplasmic reticulum [41], was employed. The addition of thapsigargin during the PGL or PGPC-induced Ca^2+^ peak, and vice versa, suggesting that PGL and PGPC did not seem to act on Ca^2+^-ATPase.

## Conclusion

We can state that both PM polyphenols and lipids exhibited an immunosuppressive effects. The PM polyphenols seem to be more active than lipids. Two molecular mechanisms seem to be involved in the immunosuppressive activity of PM extracts: i) the sustained increases in intracellular free Ca^2+^ concentration and ii) the inhibition of IL-2 mRNA expression and MAP kinase phosphorylation. Our results argue for the use of millet diet as dietary supplements for treatment of diseases associated with a sustained activation of the immune system such as autoimmune diseases.

## Abbreviations

[Ca^2+^]_i_: Free intracellular calcium concentration
EGCG: Epigallocatechin-3 gallate
ER: Endoplasmic reticulum
ERK: Extracellular signal-regulated kinase
FAMEs: Fatty acid methyl esters
GAE: Gallic acid equivalents
IFN: Interferon
IL-2: Interleukin-2
LA: Linolenic acid
MAPK: Mitogen-activated protein kinase
n-3 PUFA: Polyunsaturated fatty acids of the n-3 family
n-6 PUFA: Polyunsaturated fatty acids of the n-6 family. PBS, Phosphate-buffered saline, pH 7.4
PGL: *Pennisetum glaucum* lipids
PGPC: *Pennisetum glaucum* phenolic compounds
PKC: Protein kinase C
PM: Pearl millet
TCR: T-cell receptor
TG: Thapsigargin
